# Features of the behavior of 4-amino-5-carboxamido-1,2,3-triazole in multicomponent heterocyclizations with carbonyl compounds

**DOI:** 10.3762/bjoc.8.236

**Published:** 2012-11-30

**Authors:** Eugene S Gladkov, Katerina A Gura, Svetlana M Sirko, Sergey M Desenko, Ulrich Groth, Valentin A Chebanov

**Affiliations:** 1 Division of Chemistry of Functional Materials, State Scientific Institution “Institute for Single Crystals” of National Academy of Sciences of Ukraine, Lenin Ave. 60, 61158 Kharkiv, Ukraine; 2 Fachbereich Chemie, Universität Konstanz, Fach M-720, Universitätsstrasse 10, 78457 Konstanz, Germany; 3 Chemistry Faculty, Karazin Kharkiv National University, Svobody sq., 4, 61077 Kharkiv, Ukraine

**Keywords:** 4-amino-5-carboxamido-1,2,3-triazole, heterocycle, microwave-assisted synthesis, multicomponent reaction, ultrasound-assisted synthesis

## Abstract

Multicomponent reactions involving polyfunctional 4-amino-5-carboxamido-1,2,3-triazole and cyclic carbonyl-containing CH-acids were studied under conventional thermal heating, microwave and ultrasonic irradiation. The features of the reactions studied were discussed and the optimized procedures for the synthesis of final triazolopyrimidines were elaborated. In contrast to the similar MCRs of numerous other aminoazoles, a change of direction of the heterocyclizations in the case of 4-amino-5-carboxamido-1,2,3-triazole was not observed when microwave or thermal heating was substituted by ultrasonication at ambient temperature.

## Introduction

Multicomponent reactions (MCRs) [1–4] involving polyfunctional aminazoles as a key reagent are challenging objectives in the modern chemistry of heterocyclic compounds dealing with the synthesis of partially hydrogenated azoloazines [5–8]. The possibility of realization of several independent sequences of two-component stages of MCRs and the presence of alternative nucleophilic reaction centers in an aminazole molecule often allows the synthesis of different final heterocyclic systems from the same building blocks. However, one of the main problems preventing efficient application of polyfunctional starting materials in MCRs is the existence of simultaneous competing reactions leading to complicated mixtures of several compounds. Thereby, the development of a strategy for the control of the MCR direction and the elaboration of synthetic methods allowing tuning of their selectivity are the current objectives that have attracted our attention and efforts over the past few years [5–8].

**Figure 1:**
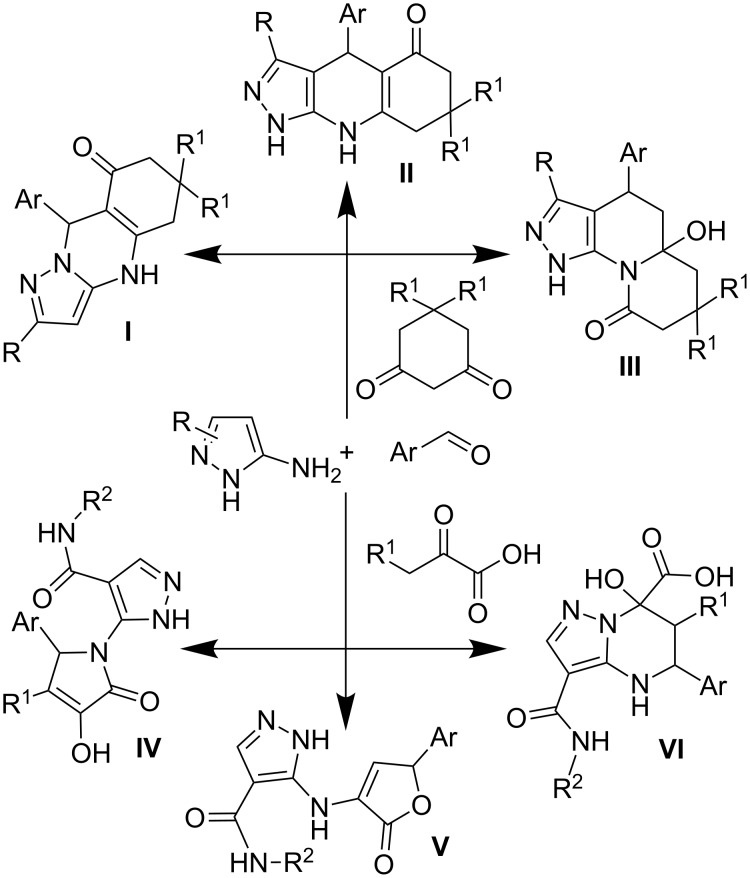
Some MCRs of aminoazoles with controlled switching of the direction.

In some of our recent publications it was demonstrated that the direction of MCRs involving aminazoles, carbonyls and active methylene compounds can be effectively controlled by variation of such reaction parameters as temperature, solvent type, catalytic system, method of activation, etc. [9–17]. Changing these parameters gives us the opportunity to synthesize different types of heterocyclic systems, with a high level of selectivity, from the same starting materials. For example, MCRs or 5-aminopyrazoles containing several nonequivalent nucleophilic reaction centers with aromatic aldehydes and derivatives of 1,3-cyclohexanedione [10–11] or pyruvic acids [12–14] can be selectively directed to the formation of one of the compounds **I**–**III** or **IV**–**VI** (Figure 1). Such an approach is in good correspondence with the “ideal synthesis” concept [18–19] and allows significant increasing of the molecular diversity of target azoloazine systems.

Among the polyfunctional aminoazoles that have been studied in heterocyclization reactions [5–8,20], derivatives of 4-amino-1,2,3-triazole have been less well investigated. There are only a few examples of their heterocyclizations with β-diketones [21], *N*-cyanomethaneimidates [22], isocyanates [23], chalcones [24] and derivatives of carboxylic acids [25–26]. The only MCR involving this type of aminoazole was described in our previous publication [27].

**Figure 2:**
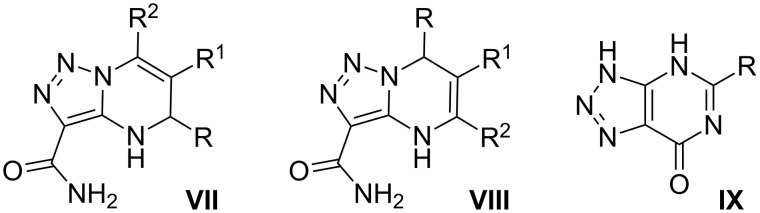
Some possible products of MCRs involving 4-amino-5-carboxamido-1,2,3-triazole and carbonyl compounds.

In the case of 4-amino-5-carboxamido-1,2,3-triazole, heterocyclizations can proceed in two main ways: “classical” for α-aminoazole direction with participation of the NH_2_-group and endocyclic NH (Figure 2, compound **VII** or its position isomer **VIII**), or an alternative pathway involving the NH_2_-group and carboxamide fragment [25–26] (compound **IX**).

Thus, there are several alternative directions for reactions between 4-amino-5-carboxamido-1,2,3-triazole, carbonyls and active methylene compounds. In the present article we disclose our recent results of a study of such MCRs and analyze the influence of some of the reaction parameters on their development and outcome.

## Results and Discussion

**Scheme 1:**
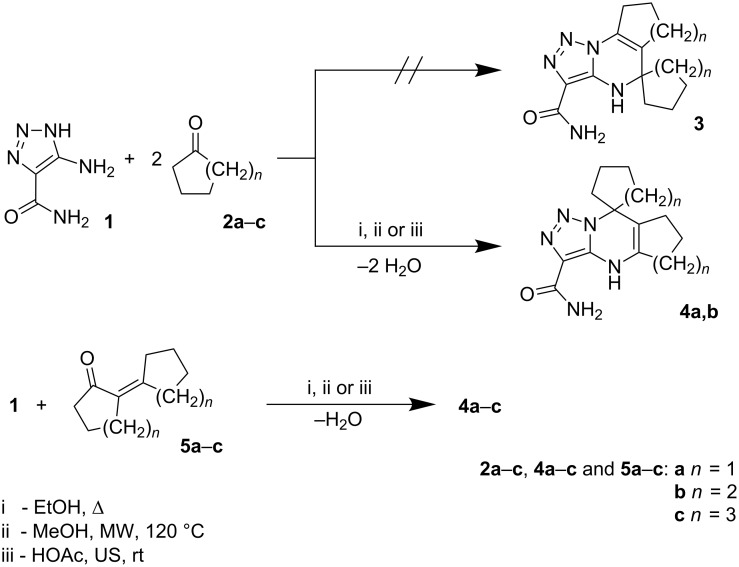
Multicomponent and sequential reactions of 4-amino-5-carboxamido-1,2,3-triazole with cyclic ketones.

It was found that reaction between one equivalent of aminotriazole **1** and two equivalents of cycloalkanones **2a,b** (*n* = 1–2) in boiling dry ethanol for 3 h (Method A) proceeded as ABB’-type [28] MCR with chemodifferentiation of cyclic ketone and yielded spiroheterocycles **4a,b** (31–37%, Scheme 1, Table 1) as the sole reaction products. However, better yields and purity of compounds **4a,b** were observed when the starting materials **1** and **2a,b** were treated in methanol under microwave (MW) irradiation at 120 °C for 30 min (Method B). At the same time, the reaction involving cycloheptanone (*n* = 3) did not allow the synthesis of compound **4c**, which was not found in the reaction mixture even in trace amounts by TLC, while aminotriazole **1** was isolated unchanged from the reaction mixture.

**Table 1:** Multicomponent synthesis of 4,5,6,7,8,9-hexahydro[1,2,3]triazolo[5,1-*b*]quinazoline-3-carboxamides **4a**–**c**, **7** and **9a**–**j**.

Entry	Aldehyde		Ketone		Product	Method^a^	Yield, %

	No	R	No	R^1^			

**1**	–	–	**5a**	–	**4a**	A (D)	31 (67)
**2**	–	–	**5b**	–	**4b**	A (B,C,D)	37 (45,27,81)
**3**	–	–	**5c**	–	**4c**	D	45
**4**	**6a**	C_6_H_5_	**2b**	–	**7**	A (B,C,D)	21 (41,60,71)
**5**	**6a**	C_6_H_5_	**8a**	H	**9a**	A (B,C)	35 (80,61)
**6**	**6b**	4-CH_3_OC_6_H_4_	**8a**	H	**9b**	B (C)	90 (43)
**7**	**6c**	2-CH_3_OC_6_H_4_	**8a**	H	**9c**	B (C)	55 (51)
**8**	**6d**	4-BrC_6_H_4_	**8a**	H	**9d**	B (C)	70 (75)
**9**	**6e**	4-ClC_6_H_4_	**8a**	H	**9e**	B (C)	61 (69)
**10**	**6a**	C_6_H_5_	**8b**	CH_3_	**9f**	A (B,C)	37 (52,78)
**11**	**6b**	4-CH_3_OC_6_H_4_	**8b**	CH_3_	**9g**	B (C)	66 (86)
**12**	**6c**	2-CH_3_OC_6_H_4_	**8b**	CH_3_	**9h**	B (C)	51 (88)
**13**	**6d**	4-BrC_6_H_4_	**8b**	CH_3_	**9i**	B (C)	90 (91)
**14**	**6e**	4-ClC_6_H_4_	**8b**	CH_3_	**9j**	B (C)	85 (77)

^a^Method A: three-component reaction, EtOH, Δ; Method B: three-component reaction, MeOH, MW; Method C: three-component reaction, HOAc, ultrasonification (US); Method D: sequential reaction, MeOH, MW.

Positional-isomeric compounds **3**, whose formation had been expected according to the data published earlier for the similar reactions of 3-amino-1,2,4-triazole [29], was not isolated or even chromatographically detected in either of the procedures applied.

It is worth noting that in our previous works [9–17] we also showed a possibility to change the direction of similar MCRs by replacement of conventional thermal or MW heating with room temperature ultrasonication. However, treatment of the starting compounds **1** and **2a,b** under ultrasonication at ambient temperature in acetic acid, which was selected instead of methanol due to solubility problems, also yielded only heterocycles **4a,b** (Method C). The yields and purity of the compounds obtained under ultrasonication were slightly lower than in the case of Methods A and B.

Spirocompound **4c** was obtained by linear reaction via preliminary synthesis and isolation of cyclic α,β-unsaturated ketone **5c** and its further treatment with 4-amino-5-carboxamido-1,2,3-triazole (**1**) in acetic acid under ultrasonication at ambient temperature, in boiling dry ethanol or under MW heating in methanol. The same procedure also allowed us to obtain compounds **5a,b**. The best results in this case were observed when the microwave-assisted procedure (Method D) was applied.

It should be noted that for similar treatments involving 3-amino-1,2,4-triazole [7,29–30] the structures of the final products were different depending on whether a multicomponent or sequential procedure was used, while in our case both procedures gave the same compounds.

**Scheme 2:**
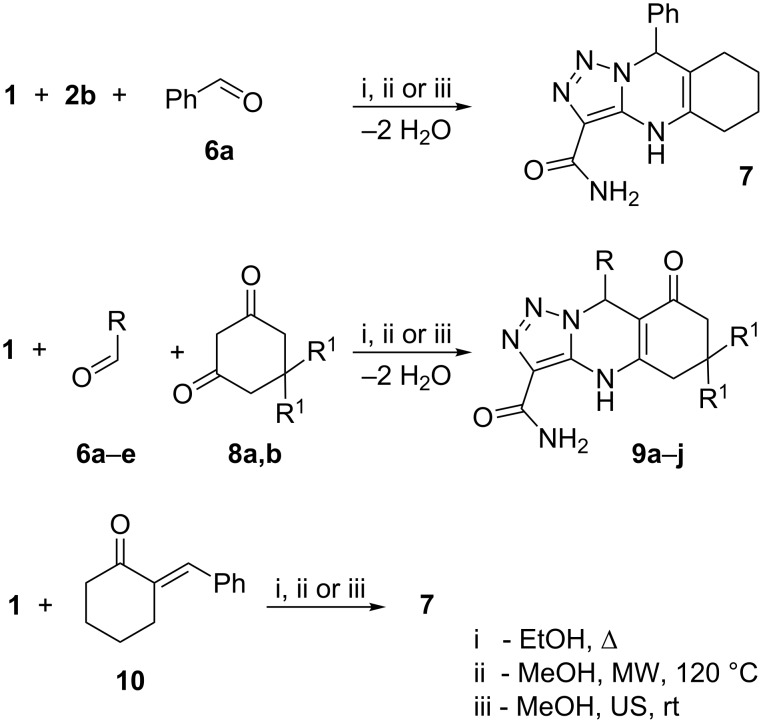
Multicomponent and sequential reactions of 4-amino-5-carboxamido-1,2,3-triazole with aldehydes and cyclic ketones.

ABC-type MCR of aminoazole **1**, cyclohexaneone **2b** and benzaldehyde **6a** was carried out in dry ethanol by conventional heating under reflux (Method A), in methanol under microwave irradiation at 120 °C (Method B), or by ultrasonication at ambient temperature in acetic acid (Method C). In all these cases triazolopyrimidine **7** was isolated as the sole reaction product (Scheme 2, Table 1). In most cases the best results from the viewpoint of yields and purity of the target compounds were observed when ultrasound-assisted method C was applied.

Sequential reaction via synthesis of arylidenecyclohexanone **10** also gave as sole reaction product compound **7** under all the methods of activation applied. However, microwave heating (Method D) was the procedure of choice in this case as well.

Multicomponent heterocyclizations of aminotriazole **1** with aromatic aldehydes **6a**–**e** and cyclohexanediones **8a,b** carried out according to Method A, B or C yielded tricyclic compounds **9a**–**j** in 51–91% (Scheme 2, Table 1). The most efficient synthetic procedure was the treatment of the starting materials in methanol under ultrasonication at ambient temperature. Very similar results were observed for MW-assisted syntheses.

The structures of the compounds synthesized were established by MS and NMR spectroscopy in combination with elemental analyses. However, MS data obtained for the reaction product isolated from the MCR between aminoazole **1** and cycloalkanone **2** may correspond to both structure **3** and **4**. Moreover, ^1^H and ^13^C NMR spectra of these compounds should contain very similar signals, and we thus used the position of the signal of pyrimidine NH in the ^1^H NMR spectra, which strongly depends on type of the structure: for angular compounds such as **3** its signal appears at 5–6 ppm while for heterocycles **4** it should be at 7–8 ppm [8,29,31–32], as was observed in our case. Additionally, the HMBC spectrum of the heterocycles obtained does not contain cross-peaks between pyrimidine NH and spiro-carbon, which is also in good correlation with structure **4**. Analogous elucidations allowed the structures of compounds **7** and **9** to be established.

## Conclusion

In summary, two types of heterocyclization reactions involving 4-amino-5-carboxamido-1,2,3-triazole and cyclic ketones were studied under conventional thermal heating, and microwave and ultrasonic irradiation. ABB’-type MCR proceeding with chemodifferentiation of cyclopentanone or cyclohexanone molecules yields 4,5,6,7-tetrahydrospiro{cyclopenta[*d*][1,2,3]triazolo[1,5-*a*]pyrimidine-8,1'-cyclopentane}-3-carboxamide (**4a**) or 5,6,7,8-tetrahydro-4*H*-spiro{[1,2,3]triazolo[5,1-*b*]quinazoline-9,1'-cyclohexane}-3-carboxamide (**4b**) under all the conditions tested. The best results were observed in the case of microwave-assisted synthesis in methanol at 120 °C. Spirocompound **4c** may be obtained by a sequential procedure via the preliminary synthesis of 2-cycloheptylidenecycloheptanone but not by a three-component reaction involving cycloheptanone. ABC-type MCR between 4-amino-5-carboxamido-1,2,3-triazole, aldehydes and cyclohexanone or 1,3-cyclohexandiones leads to the formation of 4,5,6,7,8,9-hexahydro[1,2,3]triazolo[5,1-*b*]quinazoline-3-carboxamides **7** or **9** under all the reactions conditions studied; however, the ultrasound-assisted procedure is the method of choice for this heterocyclization.

For all the MCRs studied, changes in the directions of the heterocyclizations were not observed when microwave or thermal heating was substituted by ultrasonication at ambient temperature or when the three-component reaction was replaced by a sequential protocol via the preliminary synthesis of cyclic α,β-unsaturated ketones.

## Supporting Information

File 1 Experimental details and characterization data for all new compounds.
