# Supplementary material for: Features of the behavior of 4-amino-5-carboxamido-1,2,3-triazole in multicomponent heterocyclizations with carbonyl compounds
Source: Beilstein J Org Chem. 2012 Nov 30;8:2100–5. doi: 10.3762/bjoc.8.236 (PMC3520566; doi:10.3762/bjoc.8.236)
Supplement: File 1 — Experimental details and characterization data for all new compounds. [file Beilstein_J_Org_Chem-08-2100-s001.pdf]

Supporting Information

for

Features of the behavior of 4-amino-5-carboxamido-1,2,3-triazole in multicomponent heterocyclizations with carbonyl compounds

Eugene S. Gladkov[1,2,*], Katerina A. Gura[2], Svetlana M. Sirko[1], Sergey M. Desenko[1,3], Ulrich Groth[2] and Valentin A. Chebanov[1,3]

Address: [1]Division of Chemistry of Functional Materials, State Scientific Institution "Institute for Single Crystals" of National Academy of Sciences of Ukraine, Lenin Ave. 60, 61158 Kharkiv, Ukraine, [2]Fachbereich Chemie, Universität Konstanz, Fach M-720, Universitätsstrasse 10, 78457 Konstanz, Germany and [3]Chemistry Faculty, Karazin Kharkiv National University, Svobody sq., 4, 61077 Kharkiv, Ukraine

Email: Eugene S. Gladkov[*] - gladkov@isc.kharkov.com

Experimental details and characterization data for all new compounds

1. Apparatus and analysis

Melting points of all compounds synthesized were determined with a Gallenkamp melting-point apparatus in open capillary tubes. Analytical HPLC was performed on Merck RP-18 column (250 × 4.1 mm) by using gradient or isocratic elution with an acetonitrile-water mixture (UV detection at 254 nm). The NMR spectra were recorded in DMSO-d_6 at 400 MHz (100 for ^{13}C) with Jeol Lambda 400 or Bruker Avance 400 (400 MHz) spectrometers. Low-

resolution mass spectra were measured on a GC–MS Varian 1200L (ionizing voltage 70 eV) instrument. Elemental analysis was realized on a EuroVector EA-3000. TLC analyses were performed on precoated (silica gel 60 HF_{254}) plates.

Sonication was carried out with the help of a standard ultrasonic bath operated at 44.2 kHz, in round-bottom flasks equipped with a condenser.

Microwave experiments were performed by using the Emrys™ Initiator reactor from Biotage AB (Uppsala, Sweden) possessing a single-mode microwave cavity producing controlled irradiation at 2.45 GHz. Experiments were carried out in sealed microwave process vials by using high absorbance level settings and IR temperature monitoring. Reaction times reflect the irradiation times at the set reaction temperature (fixed hold times).

All solvents and chemicals were obtained from standard commercial vendors and were used without any further purification.

Starting materials **1** [1] and **5** **a**–**c** [2,3] are known compounds. The 2-benzylidencyclohexanone (**10**) is commercially available.

2. General procedure for the synthesis of compounds 4, 7 and 9

5,6,7,8-Tetrahydro-4*H*-spiro{[1,2,3]triazolo[5,1-*b*]quinazoline-9,1'-cyclohexane}-3-carboxamide (4b)

Three-component procedure under conventional heating (Method A): 1.27 g (10 mmol) 4-Amino-5-carboxamido-1,2,3-thriazole (**1**) and 1.96 g (20 mmol) cyclohexanone in 10 mL of dry ethanol were heated for 3 h. The mixture was cooled to 0 °C, and the precipitated solid was filtered off and air dried. Yield 1.06 g (37%).

Three-component procedure under MW irradiation (Method B): A mixture of 1.27 g (10 mmol) 4-amino-5-carboxamido-1,2,3-thriazole (**1**) and 1.96 g (20 mmol) cyclohexanone in 3 mL of methanol in a 5 mL closed MW-vial was heated for 20 min at 120 °C. The reaction

mixture was cooled to 5 °C and the precipitate formed was filtered off and washed by methanol. Recrystallization from DMF-methanol afforded 1.29 g (45%).

Three-component procedure under ultrasonication (Method C): 1.27 g (10 mmol) 4-amino-5-carboxamido-1,2,3-thriazole (**1**) and 1.96 g (20 mmol) cyclohexanone in 20 mL of glacial acetic acid were irradiated in an ultrasonic bath at 35 °C for 90 min. The reaction mixture was mixed with 10 mL of water, and the precipitated solid was filtered off, washed with a small amount of cold methanol and air dried. Yield 0.76 g (27%).

Sequential procedure under MW irradiation (Method D): Mixture of 1.27 g (10 mmol) 4-amino-5-carboxamido-1,2,3-thriazole (**1**) and 1.78 g (10 mmol) cyclohexylidenecyclohexanone in 3 mL of methanol in 5 mL closed MW-vial was heated for 20 min at 120 °C . The reaction mixture was cooled to 5 °C and the precipitate formed was filtered off and washed by methanol. Recrystallization from DMF-methanol afforded 2.32 g (81%).

Mp 247–248 °C; ^{1}H NMR (400 MHz, DMSO-d_6) δ 1.30 (m, 1H, CH_2), 1.60 (m, 6H, CH_2), 1.77 (m, 3H, CH_2), 1.91 (m, 2H, CH_2), 2.02 (m, 2H, CH_2), 2.13 (m, 2H, CH_2), 2.23 (m, 2H, CH_2), 7.20 (bs, 1H, NH_2), 7.51 (bs, 1H, NH_2), 8.95 (bs, 1H, NH) ppm; ^{13}C NMR (100 MHz, DMSO-d_6) δ 26.9, 27.3, 28.2, 28.7, 29.6, 31.4, 39.1, 67.1, 111.9, 125.8, 133.6, 143.0, 168.6 ppm; Anal. calcd for $C_{15}H_{21}N_5O$ (%): C, 62.70; H, 7.37; N, 24.37; found: C, 62.74; H, 7.40; N, 24.39; Calcd MS for $[M + 1]^+$ 287.2; found: 288.3 ($[M + 1]^+$, 100), 153.9 (75), 136.0 (69); Exact. Mass. 287.2

4,5,6,7-Tetrahydrospiro{cyclopenta[*d*][1,2,3]triazolo[1,5-*a*]pyrimidine-8,1'-cyclopentane}-3-carboxamide (4a)

Mp 232 °C (with decomposition); ^{1}H NMR (200 MHz, DMSO-d_6) δ 1.63 (m, 2H, CH_2), 2.07 (m, 8H, CH_2), 2.38 (m, 2H, CH_2), 2.58 (m, 2H, CH_2), 7.18 (bs, 1H, NH), 7.51 (bs, 1H, NH),

9.01 (bs, 1H, NH) ppm; ^{13}C NMR (100 MHz, DMSO-d_6) δ 21.6, 26.8, 28.8, 31.2, 40.0, 69.9, 110.0, 121.5, 132.5, 138.6, 163.7 ppm; Anal. calcd for $C_{13}H_{17}N_5O$ (%): C, 60.21; H, 6.61; N, 27.01; found: C, 60.21; H, 6.67; N, 26.96; Calcd MS for $[M + 1]^+$ 260.1; found: 260.1 ($[M + 1]^+$, 100), 203.0 (56), 175.0 (98); Exact. Mass. 259.1

4,5,6,7,8,9-Hexahydrospiro{cyclohepta[*d*][1,2,3]triazolo[1,5-*a*]pyrimidine-10,1'-cycloheptane}-3-carboxamide (4c)

Mp 245–246 °C; ^1H NMR (200 MHz, DMSO-d_6) δ 1.61 (m, 13H, CH_2), 1.95 (m, 5H, CH_2), 2.34 (m, 4H, CH_2), 7.17 (bs, 1H, NH), 7.49 (bs, 1H, NH), 8.10 (bs, 1H, NH) ppm; ^{13}C NMR (100 MHz, DMSO-d_6) δ 25.8, 26.1, 26.9, 28.2, 31.4, 31.5, 32.1, 40.7, 67.0, 112.9, 121.5, 132.5, 137.7, 163.8 ppm; Anal. calcd for $C_{17}H_{25}N_5O$ (%): C, 64.73; H, 7.99; N, 22.20; found: C, 64.70; H, 8.03; N, 22.21. Calcd MS for $[M + 1]^+$ 316.2; found: 316.2 ($[M + 1]^+$, 100), 259.3 (65), 203.0 (95). Exact. Mass. 315.2

9-Phenyl-4,5,6,7,8,9-hexahydro[1,2,3]triazolo[5,1-*b*]quinazoline-3-carboxamide (7)

Three-component procedure under conventional heating (Method A): A mixture of 1.27 g (10 mmol) 4-amino-5-carboxamido-1,2,3-thriazole (**1**), 0.98 g (10 mmol) cyclohexanone and 1.06 g (10 mmol) benzaldehyde (**6a**) in 10 mL of dry ethanol was heated for 3 h. The mixture was cooled to 0 °C and the precipitated solid was filtered off and air dried. Yield 0.62 g (21%).

Three-component procedure under MW irradiation (Method B): 1.27 g (10 mmol) 4-Amino-5-carboxamido-1,2,3-thriazole (**1**), 0.98 g (10 mmol) cyclohexanone (**2b**) and 1.06 g (10 mmol) benzaldehyde in 3 mL of methanol in 5 mL closed MW-vial was heated for 20 min at 120 °C . The reaction mixture was cooled to 5 °C and the precipitate formed was filtered off and washed by methanol. Recrystallization from DMF-methanol afforded 1.21 g (41%).

Three-component procedure under ultrasonication (Method C): 1.27 g (10 mmol) 4-Amino-5-carboxamido-1,2,3-thriazole (**1**), 0.98 g (10 mmol) cyclohexanone (**2b**) and 1.06 g (10 mmol) benzaldehyde(**6a**) in 20 mL of glacial acetic acid were irradiated in an ultrasonic bath at 35 °C for 90 min. The reaction mixture was mixed with 10 mL of water and the precipitated solid was filtered off and washed by a small amount of cold methanol and air dried. Yield 1.77 g (60%).

Sequential procedure under MW irradiation (Method D): 1.27 g (10 mmol) 4-Amino-5-carboxamido-1,2,3-thriazole (**1**) and 1.86 g (10 mmol) 2-benzylidencyclohexanone (**10**) in 3 mL of methanol in a 5 mL closed MW-vial was heated for 20 min at 120 °C . The reaction mixture was cooled to 5 °C, and the precipitate formed was filtered off and washed by methanol. Recrystallization from DMF-methanol afforded 2.10 g (71%).

Mp 223–224 °C; ^{1}H NMR (400 MHz, DMSO-d_6) δ 1.51 (m, 4H, CH_2), 1.63 (m, 1H, CH_2), 1.87 (m, 1H, CH_2), 2.32 (m, 2H, CH_2), 6.06 (s, 1H, 9-H), 7.22 (m, 3H, Ar), 7.32 (m, 2H, Ar), 7.38 (bs, 1H, NH_2), 7.58 (bs, 1H, NH_2), 8.57 (bs, 1H, NH) ppm; ^{13}C NMR (100 MHz, DMSO-d_6) δ 22.5, 22.6, 25.7, 26.3, 62.6, 103.0, 122.3, 127.8, 128.7, 128.8, 129.3, 138.1, 141.2, 163.8 ppm; Anal. calcd for $C_{16}H_{17}N_5O$ (%): C, 65.07; H, 5.80; N, 23.71; found: C, 65.05; H, 5.85; N, 23.70. MS (70 eV): *m/z* = 295 (M^+, 14), 162 (78), 49 (100). Exact. Mass. 295.1

8-Oxo-9-phenyl-4,5,6,7,8,9-hexahydro[1,2,3]triazolo[5,1-*b*]quinazoline-3-carboxamide (9a)

The compounds **9a–j** were obtained according to the procedures described above for compound **7**.

Mp 314–315 °C (with decomposition); ^{1}H NMR (400 MHz, DMSO-d_6) δ 1.92 (m, 2H, CH_2), 2.28 (m, 2H, CH_2), 2.71 (m, 1H, CH_2), 2.97 (m, 1H, CH_2), 6.59 (s, 1H, 9-H), 7.27 (m, 3H,

Ar), 7.31 (m, 2H, Ar), 7.46 (bs, 1H, NH_2), 7.80 (bs, 1H, NH_2), 10.20 (bs, 1H, NH) ppm; ^{13}C NMR (100 MHz, DMSO-d_6) δ 20.6, 26.3, 36.3, 56.5, 106.3, 123.8, 126.8, 128.0, 128.5, 134.8, 141.3, 152.2, 162.3, 193.4 ppm; Anal. calcd for $C_{16}H_{15}N_5O_2$ (%): C, 62.13; H, 4.89; N, 22.64; found: C, 62.16; H, 4.95; N, 22.68. Calcd MS for $[M + 1]^+$ 310.1; found: 310.2 (M + 1, 100), 289.2 (92), 217.1 (17). Exact. Mass. 309.1

9-(4-Methoxyphenyl)-8-oxo-4,5,6,7,8,9-hexahydro[1,2,3]triazolo[5,1-*b*]quinazoline-3-carboxamide (9b)

Mp 279 °C (with decomposition); ^{1}H NMR (400 MHz, DMSO-d_6) δ 1.96 (m, 2H, CH_2), 2.29 (m, 2H, CH_2), 2.70 (m, 1H, CH_2), 2.95 (m, 1H, CH_2), 3.71 (s, 3H, OCH_3), 6.54 (s, 1H, 9-H), 6.85 (d, 2H, Ar), 7.18 (d, 2H, Ar), 7.43 (bs, 1H, NH_2), 7.78 (bs, 1H, NH_2), 10.23 (bs, 1H, NH) ppm; ^{13}C NMR (100 MHz, DMSO-d_6) δ 20.6, 26.3, 36.3, 55.1, 56.0, 106.5, 113.8, 123.8, 128.1, 133.6, 134.7, 152.0, 158.9, 162.4, 193.4 ppm; Anal. calcd for $C_{17}H_{17}N_5O_3$ (%): C, 60.17; H, 5.05; N, 20.64; found: C, 60.14; H, 5.10; N, 20.65. Calcd MS for $[M + 1]^+$ 340.1; found: 340.3 (M + 1, 100), 289.1 (81), 217.1 (93). Exact. Mass. 339.1

9-(2-Methoxyphenyl)-8-oxo-4,5,6,7,8,9-hexahydro[1,2,3]triazolo[5,1-*b*]quinazoline-3-carboxamide (9c)

Mp 282 °C (with decomposition); ^{1}H NMR (400 MHz, DMSO-d_6) δ 1.82 (m, 1H, CH_2), 1.95 (m, 1H, CH_2), 2.23 (m, 2H, CH_2), 2.69 (m, 1H, CH_2), 2.93 (m, 1H, CH_2), 3.69 (s, 3H, OCH_3), 6.70 (s, 1H, 9-H), 6.89 (t, 1H, Ar), 6.95 (d, 1H, Ar), 7.23 (t, 1H, Ar), 7.32 (d, 1H, Ar), 7.39 (bs, 1H, NH_2), 7.72 (bs, 1H, NH_2), 10.16 (bs, 1H, NH) ppm; ^{13}C NMR (100 MHz, DMSO-d_6) δ 20.7, 26.3, 36.4, 53.7, 55.8, 105.6, 112.2, 120.2, 123.2, 129.1, 129.5, 129.7, 135.4, 152.4, 157.2, 162.5, 193.1 ppm; Anal. calcd for $C_{17}H_{17}N_5O_3$ (%): C, 60.17; H, 5.05; N, 20.64; found:

C, 60.13; H, 5.11; N, 20.63. Calcd MS for $[M + 1]^+$ 340.1; found: 340.1 (M + 1, 100), 263.9 (44), 175.9 (84). Exact. Mass. 339.1

9-(4-Bromophenyl)-8-oxo-4,5,6,7,8,9-hexahydro[1,2,3]triazolo[5,1-*b*]quinazoline-3-carboxamide (9d)

Mp 279 °C (with decomposition); ^{1}H NMR (400 MHz, DMSO-d_6) δ 1.94 (m, 2H, CH_2), 2.27 (m, 2H, CH_2), 2.71 (m, 1H, CH_2), 2.94 (m, 1H, CH_2), 6.58 (s, 1H, 9-H), 7.22 (d, 2H, Ar), 7.44 (bs, 1H, NH_2), 7.50 (d, 2H, Ar), 7.81 (bs, 1H, NH_2), 10.32 (bs, 1H, NH) ppm; ^{13}C NMR (100 MHz, DMSO-d_6) δ 21.0, 26.3, 36.2, 56.1, 105.9, 123.8, 129.2, 131.4, 134.7, 140.6, 152.4, 162.3, 172.0, 193.5 ppm; Anal. calcd for $C_{16}H_{14}BrN_5O_2$ (%): C, 49.50; H, 3.63; N, 18.04; found: C, 49.48; H, 3.69; N, 18.06. Calcd MS for $[M + 1]^+$ 387.0; found: 388.2 (M + 1, 100), 307.1 (31), 289.2 (27). Exact. Mass. 387.0

9-(4-Chlorophenyl)-8-oxo-4,5,6,7,8,9-hexahydro[1,2,3]triazolo[5,1-*b*]quinazoline-3-carboxamide (9e)

Mp 268 °C (with decomposition); ^{1}H NMR (400 MHz, DMSO-d_6) δ 1.93 (m, 2H, CH_2), 2.29 (m, 2H, CH_2), 2.73 (m, 1H, CH_2), 2.95 (m, 1H, CH_2), 6.60 (s, 1H, 9-H), 7.29 (d, 2H, Ar), 7.37 (d, 2H, Ar), 7.46 (bs, 1H, NH_2), 7.82 (bs, 1H, NH_2), 10.34 (bs, 1H, NH_2) ppm; ^{13}C NMR (100 MHz, DMSO-d_6) δ 20.6, 26.3, 36.3, 56.0, 105.9, 123.9, 128.5, 128.9, 132.6, 134.8, 140.2, 152.4, 162.3, 193.4 ppm; Anal. calcd for $C_{16}H_{14}ClN_5O_2$ (%): C, 55.90; H, 4.10; N, 20.37; found: C, 55.92; H, 4.15; N, 20.37. Calcd MS for $[M + 1]^+$ 343.1; found: 344.2 (M + 1, 100), 298.1 (12), 232.1 (10). Exact. Mass. 343.1

6,6-Dimethyl-8-oxo-9-phenyl-4,5,6,7,8,9-hexahydro[1,2,3]triazolo[5,1-*b*]quinazoline-3-carboxamide (9f)

Mp 307 °C (with decomposition); ^{1}H NMR (400 MHz, DMSO-d_6) δ 0.93 (s, 3H, CH_3), 1.03 (s, 3H, CH_3), 2.16 (q, 2H, CH_2), 2.72 (q, 2H, CH_2), 6.55 (s, 1H, 9-H), 7.23 (m, 3H, Ar), 7.29 (m, 2H, Ar), 7.43 (bs, 1H, NH_2), 7.79 (bs, 1H, NH_2), 10.19 (bs, 1H, NH) ppm; ^{13}C NMR (100 MHz, DMSO-d_6) δ 26.8, 28.6, 32.1, 39.4, 49.8, 56.8, 105.4, 123.9, 126.8, 128.0, 128.5, 135.0, 141.4, 150.1, 162.3, 193.1 ppm; Anal. calcd for $C_{18}H_{19}N_5O_2$ (%): C, 64.08; H, 5.68; N, 20.76; found: C, 64.10; H, 5.71; N, 20.75. Calcd MS for $[M + 1]^+$ 338.2; found: 338.3 (M + 1, 100), 289.1 (65), 217.1 (98). Exact. Mass. 337.2

9-(4-Methoxyphenyl)-6,6-dimethyl-8-oxo-4,5,6,7,8,9-hexahydro[1,2,3]triazolo[5,1-*b*]quinazoline-3-carboxamide (9g)

Mp 283 °C (with decomposition); ^{1}H NMR (400 MHz, DMSO-d_6) δ 0.95 (s, 3H, CH_3), 1.03 (s, 3H, CH_3), 2.15 (q, 2H, CH_2), 2.71 (q, 2H, CH_2), 3.69 (s, 3H, OCH_3), 6.50 (s, 1H, 9-H), 6.84 (d, 2H, Ar), 7.15 (d, 2H, Ar), 7.42 (bs, 1H, NH_2), 7.78 (bs, 1H, NH_2), 10.14 (bs, 1H, NH) ppm; ^{13}C NMR (100 MHz, DMSO-d_6) δ 26.8, 28.6, 32.1, 39.4, 49.8, 55.1, 56.3, 105.5, 113.8, 123.8, 128.1, 133.7, 134.9, 150.0, 158.9, 162.4, 193.1 ppm; Anal. calcd for $C_{19}H_{21}N_5O_3$ (%): C, 62.11; H, 5.76; N, 19.06; found: C, 62.14; H, 5.82; N, 19.04. Calcd MS for $[M + 1]^+$ 368.2; found: 368.3 (M + 1, 100), 322.2 (63), 204.0 (71). Exact. Mass. 367.2

9-(2-Methoxyphenyl)-6,6-dimethyl-8-oxo-4,5,6,7,8,9-hexahydro[1,2,3]triazolo[5,1-*b*]quinazoline-3-carboxamide (9h)

Mp 254 °C; ^{1}H NMR (400 MHz, DMSO-d_6) δ 0.91 (s, 3H, CH_3), 1.04 (s, 3H, CH_3), 2.12 (q, 2H, CH_2), 2.69 (q, 2H, CH_2), 3.66 (s, 3H, OCH_3), 6.67 (s, 1H, 9-H), 6.91 (m, 2H, Ar), 7.24 (t, 1H, Ar), 7.34 (d, 1H, Ar), 7.39 (bs, 1H, NH_2), 7.73 (bs, 1H, NH_2), 10.09 (bs, 1H, NH) ppm;

^{13}C NMR (100 MHz, DMSO-d_6) δ 26.2, 28.9, 32.0, 39.4, 49.9, 54.3, 55.5, 104.3, 111.7, 120.0, 123.2, 128.7, 129.5, 130.0, 135.6, 150.5, 157.0, 163.6, 192.8 ppm; Anal. calcd for $C_{19}H_{21}N_5O_3$ (%): C, 62.11; H, 5.76; N, 19.06; found: C, 62.10; H, 5.81; N, 19.09. Calcd MS for $[M + 1]^+$ 368.2; found: 368.2 (M + 1, 100), 291.1 (43), 204.0 (89). Exact. Mass. 367.2

9-(4-Bromophenyl)-6,6-dimethyl-8-oxo-4,5,6,7,8,9-hexahydro[1,2,3]triazolo[5,1-*b*]quinazoline-3-carboxamide (9i)

Mp 264 °C (with decomposition); ^1H NMR (400 MHz, DMSO-d_6) δ 0.95 (s, 3H, CH_3), 1.04 (s, 3H, CH_3), 2.17 (q, 2H, CH_2), 2.73 (q, 2H, CH_2), 6.57 (s, 1H, 9-H), 7.21 (d, 2H, Ar), 7.46 (bs, 1H, NH_2), 7.52 (d, 2H, Ar), 7.83 (bs, 1H, NH_2), 10.26 (bs, 1H, NH) ppm; ^{13}C NMR (100 MHz, DMSO-d_6) δ 26.9, 28.5, 32.1, 39.4, 49.8, 56.3, 104.9, 121.2, 123.9, 129.2, 131.4, 134.9, 140.8, 150.3, 162.3, 193.1 ppm; Anal. calcd for $C_{18}H_{18}BrN_5O_2$ (%): C, 51.94; H, 4.36; N, 16.82; found: C, 51.92; H, 4.40; N, 16.83. Calcd MS for $[M + 1]^+$ 415.1; found: 416.0 (M + 1, 100), 307.2 (27), 260.1 (18). Exact. Mass. 415.1

9-(4-Chlorophenyl)-6,6-dimethyl-8-oxo-4,5,6,7,8,9-hexahydro[1,2,3]triazolo[5,1-*b*]quinazoline-3-carboxamide (9j)

Mp 266 °C (with decomposition); ^1H NMR (400 MHz, DMSO-d_6) δ 0.95 (s, 3H, CH_3), 1.04 (s, 3H, CH_3), 2.17 (q, 2H, CH_2), 2.73 (q, 2H, CH_2), 6.59 (s, 1H, 9-H), 7.27 (d, 2H, Ar), 7.38 (d, 2H, Ar), 7.46 (bs, 1H, NH_2), 7.83 (bs, 1H, NH_2), 10.26 (bs, 1H, NH) ppm; ^{13}C NMR (100 MHz, DMSO-d_6) δ 26.9, 28.5, 32.1, 39.4, 49.8, 56.2, 105.0, 123.9, 128.5, 128.8, 132.6, 134.9, 140.4, 150.3, 162.3, 193.1 ppm; Anal. calcd for $C_{18}H_{18}ClN_5O_2$ (%): C, 58.14; H, 4.88; N, 18.84; found: C, 58.16; H, 4.93; N, 18.83. Calcd MS for $[M + 1]^+$ 371.1; found: 372.3 (M + 1, 100), 326.2 (37), 273.2 (29). Exact. Mass. 371.1

References

1. Hoover, J. R. E.; Day, A. R. *J. Am. Chem. Soc.* **1956,** *78,* 5832. doi:10.1021/ja01603a033
2. Roth, H. J.; Thaßler K. *Arch. Pharm. Ber. Dtsch. Pharm. Ges.* **1971,** *304,* 824. doi:10.1002/ardp.19713041107
3. Martin A. Cyclopentylidene-cyclopentanol in pereumery. U.S. Patent 5776884, July 07, 1998.
